# Supplementary material for: Molecular and Kinetic Analyses of Circulating Tumor Cells as Predictive Markers of Treatment Response in Locally Advanced Rectal Cancer Patients
Source: Cells. 2019 Jun 26;8(7):641. doi: 10.3390/cells8070641 (PMC6679115; doi:10.3390/cells8070641)
Supplement: Supplementary file 1 [file cells-08-00641-s001.pdf]

Table 1. table of sensitivity, specificity and predictive values of biomarkers evaluated.

**Biomarker**	**Sensitivity (%)**	**Specificity (%)**	**Positive Predictive Value (%)**	**Negative Predictive Value (%)**	***p* value**
**CISH TYMS before NCRT**	83.3	83.3	95.2	55.5	0.001
**TYMS protein before NCRT**	83.3	25	85.7	21.7	0.66
**RAD23B protein before NCRT**	66.6	45.8	84.6	23.5	0.58
**CISH TYMS after NCRT**	75	68.2	93.8	30	0.102
**TYMS protein after NCRT**	100	50	100	28.6	0.064
**RAD23B protein after NCRT**	100	70	100	40	0.01
